# Every Third Male Patient with Acromegaly Recovers from Hypogonadism after Neurosurgical Treatment

**DOI:** 10.3390/jcm13185526

**Published:** 2024-09-18

**Authors:** Aleksandra Derwich-Rudowicz, Kacper Nijakowski, Aleksandra Biczysko, Katarzyna Ziemnicka, Włodzimierz Liebert, Marek Ruchała, Nadia Sawicka-Gutaj

**Affiliations:** 1 Department of Endocrinology, Metabolism and Internal Medicine, Poznan University of Medical Sciences, 60-355 Poznań, Poland; 2 Department of Conservative Dentistry and Endodontics, Poznan University of Medical Science, 60-355 Poznań, Poland; 3 Department of Neurosurgery, Poznan University of Medical Science, 60-355 Poznań, Poland

**Keywords:** GH, testosterone, hypogonadotropic hypogonadism, acromegaly, pituitary tumor, pituitary adenoma

## Abstract

**Background:** Acromegaly is a rare endocrine condition caused by excessive growth hormone (GH) production. Hypogonadotropic hypogonadism (HH) affects 30%–50% of acromegaly patients. **Objectives:** This study examined the frequency of HH in men with acromegaly and the effects of neurosurgical treatment during the follow-up period. **Materials and Methods:** A retrospective analysis of medical records from January 2015 to December 2022 was conducted. Data included clinical history, laboratory results, and pituitary MRI findings. Statistical analysis was performed using Statistica 13.3. **Results:** Patients were divided into two groups: a cross-sectional sample (preoperative n = 62; postoperative n = 60) and a longitudinal sample (n = 53). In the longitudinal sample, preoperative HH was diagnosed in 41 males (77.36%). Post-surgery, HH prevalence decreased to 58.49% (n = 31), with a significant increase in postoperative testosterone levels (9.1 vs. 12.1 nmol/L; *p* < 0.001), particularly in patients with preoperative HH (7.2 vs. 10.2 nmol/L; *p* < 0.001). Among 41 patients with HH, 12 (29.27%) showed recovery. Testosterone levels were lower in patients with macroadenomas (7.2 nmol/L vs. 11.05 nmol/L; *p* < 0.001). Patients with HH had higher baseline levels of GH and insulin-like growth factor 1 (IGF-1) (GH: 3.37 ng/mL; IGF-1: 551 ng/mL vs. GH: 1.36 ng/mL; IGF-1: 355 ng/mL). Luteinizing hormone (LH) levels above 3.3 mIU/mL and follicle-stimulating hormone (FSH) levels above 4.4 mIU/mL predicted hypogonadism remission (Area under the curve (AUC): 0.838 and 0.792, respectively). **Conclusions:** Younger patients with macroadenoma and hyperprolactinemia are more likely to have preoperative hypogonadism. Neurosurgical treatment can normalize LH, FSH, and total testosterone in approximately 30% of these patients.

## 1. Introduction

Acromegaly is a rare disease caused by a chronic excess of growth hormones (GH), mainly caused by a pituitary adenoma [1]. The estimated prevalence of the disease is 36–60 cases/1,000,000 in the population [1]. Excess secretion of GH causes the overproduction of insulin-like growth factor 1 (IGF-1), which exerts somatic and metabolic effects. This includes the stimulation of growth in various tissues, such as bone, skin, connective tissue, bone, viscera, cartilage, and numerous epithelial tissues [1,2,3]. The metabolic effects encompass nitrogen retention, insulin antagonism, and lipolysis [3]. Patients with acromegaly have an increased risk of developing cardiovascular diseases (CVD), with atrial hypertension being present in almost 40% of cases; certain tumors; sleep apnea and obstruction of upper airways; visceromegaly; osteoarthritis; and diabetes mellitus [1,2,3].

Hypogonadotropic hypogonadism (HH) is present in up to 50% of acromegaly patients [4,5]. A strong correlation exists between the somatotropic axis and gonadal function. The presence of pituitary macroadenomas can lead to the suppression of secretion of follicle-stimulating hormones (FSH) and luteinizing hormones (LH), either by directly compressing gonadotropic cells or by causing deviation in the pituitary stalk, resulting in hyperprolactinemia. Conversely, elevated GH and IGF-1 disrupt the normal pulsatile pattern of gonadotropin secretion at the hypothalamic–pituitary level [6]. Decreased LH corresponds with diminished stimulation of testicular Leydig cells, leading to hypogonadism. Reduced FSH is associated with reduced activity in Sertoli cells, essential supporters of germ cells [7]. Ultimately, hypogonadism may also be the consequence of surgical treatment or radiotherapy, as hypopituitarism is one of the most common complications, affecting up to 30% percent of patients. Impaired testicular function, abnormal spermatogenesis, and decreased serum testosterone levels result in male infertility, decreased libido, erectile dysfunction, and loss of morning erection, as well as an increase in body mass index (BMI) and reduced physical strength in these patients [8]. The objective of this study was to examine the frequency of hypogonadism in male patients with acromegaly and assess the impact of surgery on the pituitary–testicular axis during the follow-up period.

## 2. Materials and Methods

**Figure 1:**
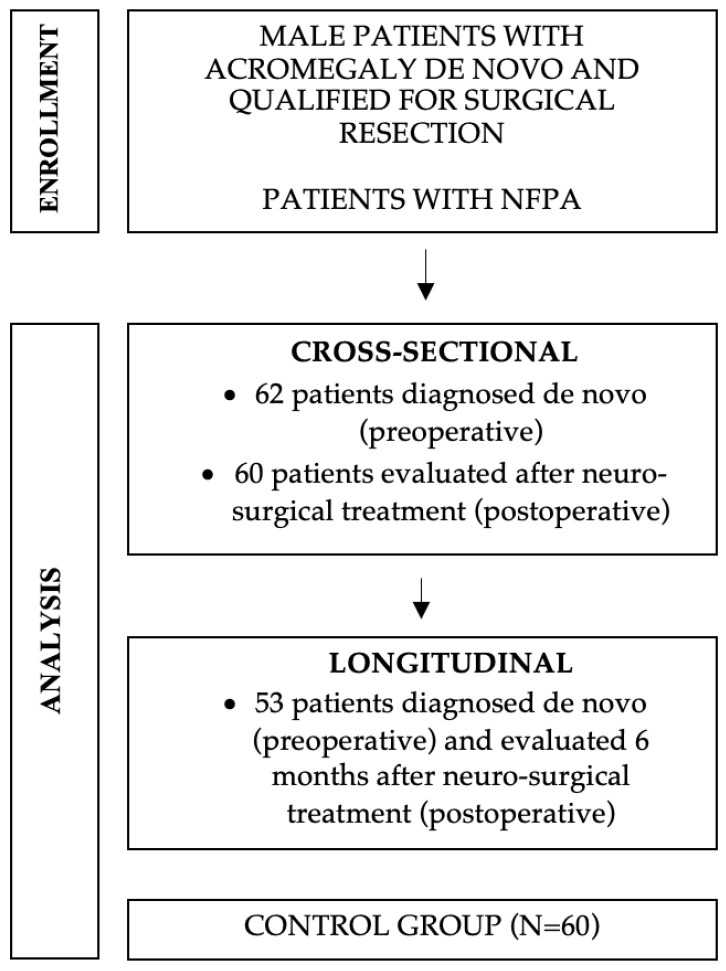
Flow chart of the study.The diagnosis of acromegaly was made when all the criteria were fulfilled: (1) elevation of IGF-1 above the age-adjusted upper normal range; (2) nadir GH above 1 ng/mL in a 75 g oral glucose tolerance test (patients without diabetes) or random GH levels above 2.5 ng/mL (mean of five measurements repeated every 30 min in patients with diabetes) [9,10,14,15]; and (3) a pituitary gland tumor detected in magnetic resonance imaging (MRI) [3].

The study had a retrospective design. Medical records for all male patients diagnosed with acromegaly, hospitalized pre- and postoperatively in the Department of Endocrinology, Metabolism and Internal Medicine at the Poznan University of Medical Sciences in Poland between January 2015 and December 2022 have been analyzed. We divided patients into two groups: the cross-sectional sample (preoperative n = 62, and postoperative n = 60) and the longitudinal sample (n = 53), which was separated from the cross-sectional group and comprised patients diagnosed with acromegaly de novo and evaluated up to 6 months after neurosurgical treatment. The study flow chart is presented in Figure 1. Before surgery patients received lanreotide 120 mg subcutaneously for 3 months. The samples are described in Table 1. All enrolled patients received pituitary tumor resection surgery. Routine postoperative follow-up, which began 3 months after surgery, was scheduled for all patients. Clinical history, laboratory results, GH, IGF-1, LH, FSH, testosterone, prolactin, sex hormone binding globulin (SHBG), free testosterone index (FTI), complete blood count, prostate specific antigen (PSA), glucose, lipid profile, and endocrine tests were routinely recorded. Age, gender, diagnostic images, and BMI were also reviewed and documented. A detailed medical history was taken. All parameters were measured in blood samples taken after overnight fasting. Acromegaly and hypogonadism were diagnosed and treated according to the current guidelines [9,10,11,12,13]. The assays were performed using the method according to the manufacturer’s recommendations. Testosterone, LH, FSH, and SHBG were measured via electrochemiluminescence (ECLIA) using the Cobas e801 analyzer (Roche Diagnostics, Indianapolis, IN, USA). FTI was calculated from the ratio of total testosterone (TT) to SHBG [% FTI = (100 TT/SHBG)], the bioavailable testosterone, was calculated via the association constant to albumin. The GH levels were assayed via ECLIA using the Cobas e402 analyzer (Roche Diagnostics). IGF-1 levels were measured via the chemiluminescence (CMIA) method using a LIAISON Analyzer (DiaSorin Ltd., Saluggia, Italy), reference intervals (95% CI) according to age range (ng/mL), and specifications of the certified laboratory: (a) 18–20 y 186–453; (b) 21–23 y 168–411; (c) 24–26 y 153–377; (d) 27–29 y 142–351; (e) 30–39 y 124–310; (f) 40–49 y 106–271; (g) 50–59 y 97–252; (h) 60–69 y 92–245; (i) 70–89 y 80–220). Total testosterone (TT) concentrations were measured in the morning after overnight fasting. Diagnosis of hypogonadism was made if the measured TT was <12 nmol/L with concomitant symptoms. In patients with TT in the range of 8–12 nmol/L, we performed LH-releasing hormone (LHRH) stimulation. Blood samples were obtained while participants were in the supine position and after an overnight fast, starting at 8:00 a.m. Blood samples were drawn from a catheter positioned in an antecubital vein at 0, 30, 60, and 120 min after stimulation. In the absence of at least a 2-fold increase in LH and a 1.5-fold increase in FSH after 30–45 min, HH was diagnosed. Prolactin was measured via ECLIA using the Cobas e801 analyzer (Roche Diagnostics, Indianapolis, IN, USA), N: 85–390 μIU/mL. Hyperprolactinemia was diagnosed when elevated PRL levels were detected (samples were treated with polyethylene glycol (PEG) to precipitate macroprolactin and then re-measured). Nadir GH was measured after a 75 g glucose tolerance test. In diabetic patients, serum GH levels were determined every 30 min and the arithmetical mean was counted based on five measurements [9,10,14,15]. All patients underwent an MRI (magnetic resonance imaging) scan of the pituitary gland to determine image characteristics: tumor size, intratumor hemorrhage, and invasion type (MRI: Siemens Magnetom Avanto, serial number 26184, to 2017, and Siemens Magnetom Skyra, serial number 145114, from 2017). The greatest diameter of the tumor was measured as the tumor size. Macroadenoma was defined as a tumor with a diameter ≥ 10 mm. The control group for cross-sectional sampling consisted of male patients with non-functioning pituitary adenoma (NFPA) matched for age and tumor size, hospitalized in the Department of Endocrinology, Metabolism and Internal Medicine at the Poznan University of Medical Sciences in Poland between January 2015 and December 2022.

The SAGIT instrument was completed using patients’ medical records, providing a comprehensive evaluation of key components associated with acromegaly: signs and symptoms (S), associated comorbidities (A), GH levels (G), IGF-1 levels (I), and tumor features (T). Each component was scored on a scale of zero up to a maximum value (S: 0–4, A: 0–6, G: 0–4, I: 0–3, and T: 0–5). Higher scores in individual categories and the total sum of points indicated greater advancement of the respective factor and overall disease activity [16,17,18].

This study was approved by the Bioethical Committee of the Poznan University of Medical Sciences, with informed consent waived due to its retrospective nature (Decision No. 633/22) [18]. All methods adhered to relevant guidelines and regulations [19].

Descriptive statistics of quantitative variables were expressed in medians. Due to non-compliance with the normal distribution, quantitative variables were compared using the Wilcoxon test or the Mann–Whitney test. Qualitative variables were compared using Pearson’s Chi-square test. Also, Spearman’s correlation coefficients were determined between parameters for testosterone and diagnostic parameters for hypogonadism and acromegaly. The comparison between groups was performed with Kruskal–Wallis test with Dunn post-hoc. Receiver operating characteristic (ROC) analysis was performed to assess predictive values for LH and FSH. A significance level of α = 0.05 was applied to all analyses. Statistical analysis was performed using Statistica 13.3 (StatSoft, Cracow, Poland).

## 3. Results

The cross-sectional analysis involved a total of 62 pre-operative and 60 post-operative male patients aged 22–76 years. The longitudinal analysis compared 53 male patients aged 24–88 years at the time of diagnosis and after neurosurgical treatment. The control group for cross-sectional sampling consisted of 60 male patients aged 18–80 with NFPA. The clinical characteristics of all cohorts are summarized in Table 1.

### 3.1. Characteristics of the Cross-Sectional Sample

**Figure 2:**
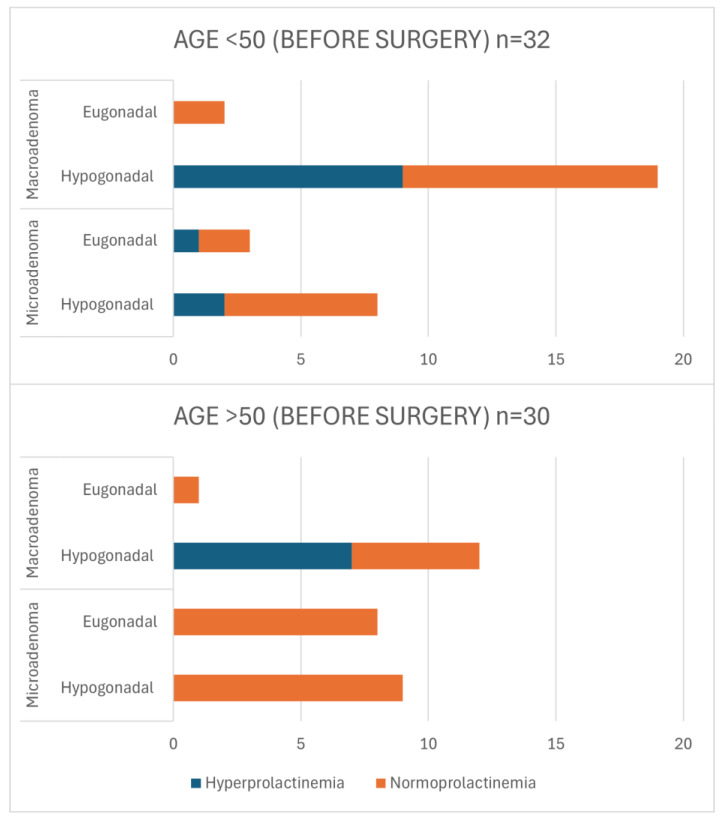
Characteristics of pre-operative patients in terms of age, tumor size, gonadal status, and presence of hyperprolactinemia in cross-sectional sample. *p*-values for Chi-square test (<50 vs. >50): eu- vs. hypogonadism *p* = 0.176; micro- vs. macroadenoma *p* = 0.078; normo- vs. hyperprolactinemia *p* = 0.227.

The results of further analysis of the cross-sectional sample, focusing on patients aged <50 and >50 years, provide a detailed understanding of pre- and post-operative characteristics, particularly in relation to the tumor size, gonadal status, and the presence of hyperprolactinemia. Figure 2 outlines the pre-operative characteristics for both age groups (and detailed data are presented in Supplementary Table S1). Hypogonadism at the time of diagnosis was manifested more frequently in younger patients (<50 years; 84% vs. 70%, *p* = 0.176), as they were diagnosed with larger tumors (*p* = 0.078) and with concurrent hyperprolactinemia (*p* = 0.227). However, none of these differences reached statistical significance. For patients aged <50 years, the majority (65%) were diagnosed with macroadenomas, and among these, a substantial 90% had hypogonadism. Hyperprolactinemia was present in 47% of these patients. In contrast, among those with microadenomas in this age group, 73% were diagnosed with hypogonadism, and 25% of cases demonstrated concurrent hyperprolactinemia. In the >50 years age group, 43% of patients were diagnosed with macroadenomas, with 92% of these presenting with hypogonadism. Hyperprolactinemia was identified in 58% of these patients. Among those with microadenomas in this age category, 53% were diagnosed with hypogonadism.

**Figure 3:**
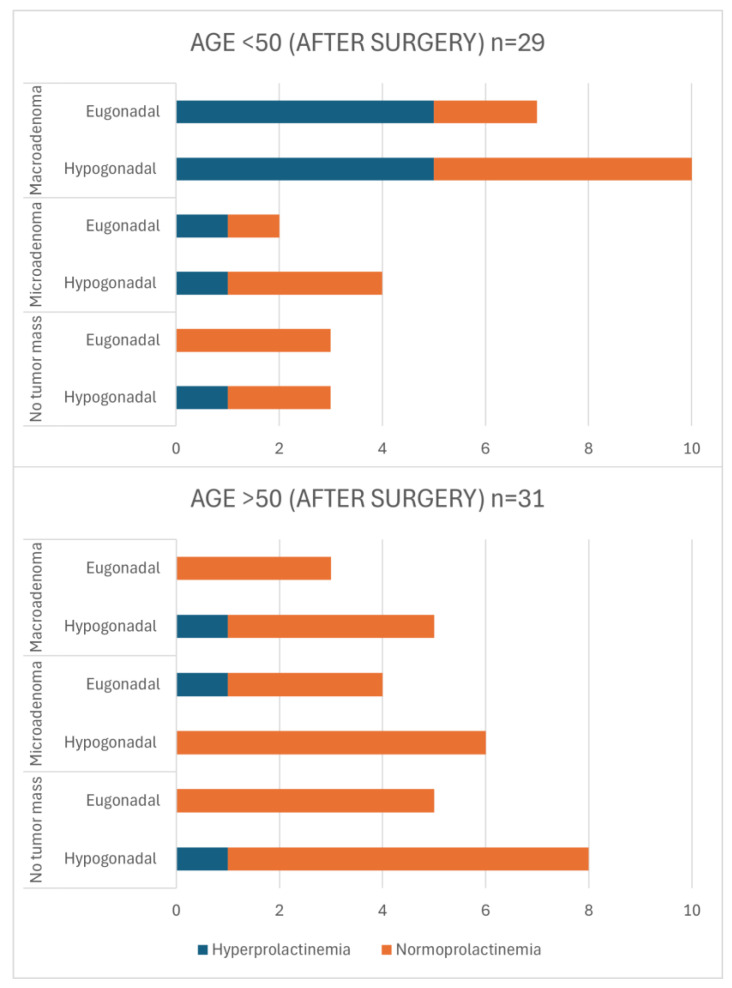
Characteristics of post-operative patients in terms of age, tumor size, gonadal status, and presence of hyperprolactinemia in cross-sectional sample. *p*-values for Chi-square test (<50 vs. >50): eu- vs. hypogonadism *p* = 0.834; micro- vs. macroadenoma *p* = 0.010; normo- vs. hyperprolactinemia *p* = 0.002.

Figure 3 provides an overview of the post-operative group (a detailed characterization of patients in this group is also presented in Supplementary Table S2). These data reveal the hypogonadism in individuals under 50 years old, which was particularly noteworthy in cases where no tumor mass was detected. Among this subgroup, three out of six patients exhibited hypogonadism, with one case additionally displaying concurrent hyperprolactinemia. For patients in the same age group with microadenomas, four out of six individuals experienced hypogonadism. Among patients with macroadenomas, 10 out of 17 patients were hypogonadal after surgery. In the older age group (>50 years), 13 patients were diagnosed with no tumor mass in MRI, and of these, 8 were found to be hypogonadal. Additionally, hyperprolactinemia was present in one patient. Among individuals with microadenomas, 6 out of 10 patients presented with hypogonadism. Similarly, in patients with macroadenomas, 5 out of 8 individuals were diagnosed with hypogonadism. The comparison between patients <50 and >50 years old with eugonadism and hypogonadism revealed no statistically significant difference (*p* = 0.834). However, the analysis showed significant differences when comparing patients with microadenomas versus macroadenomas (*p* = 0.010) and those with normoprolactinemia versus hyperprolactinemia (*p* = 0.002).

#### Differences between Micro- and Macroadenomas

We compared patients who presented with micro- and macroadenomas at the time of diagnosis. We also compared patients with acromegaly to those with non-functioning pituitary adenomas, matched for age and adenoma size (control group-CG). Testosterone levels were found to be statistically significantly lower in patients with macroadenoma compared to microadenoma in the cross-sectional sample (7.2 nmol/L vs. 11.05 nmol/L; *p* < 0.001). CG. Prolactin levels were also found to be elevated in patients with hypogonadism and macroadenoma (304 μIU/mL; *p* = 0.033). GH and IGF-1 concentrations were significantly higher in patients with macroadenoma (*p* < 0.001). Comparisons of laboratory parameters before neurosurgical treatment in patients with micro- and macroadenoma is presented in Table 2.

### 3.2. Characteristics of the Longitudinal Sample

**Figure 4:**
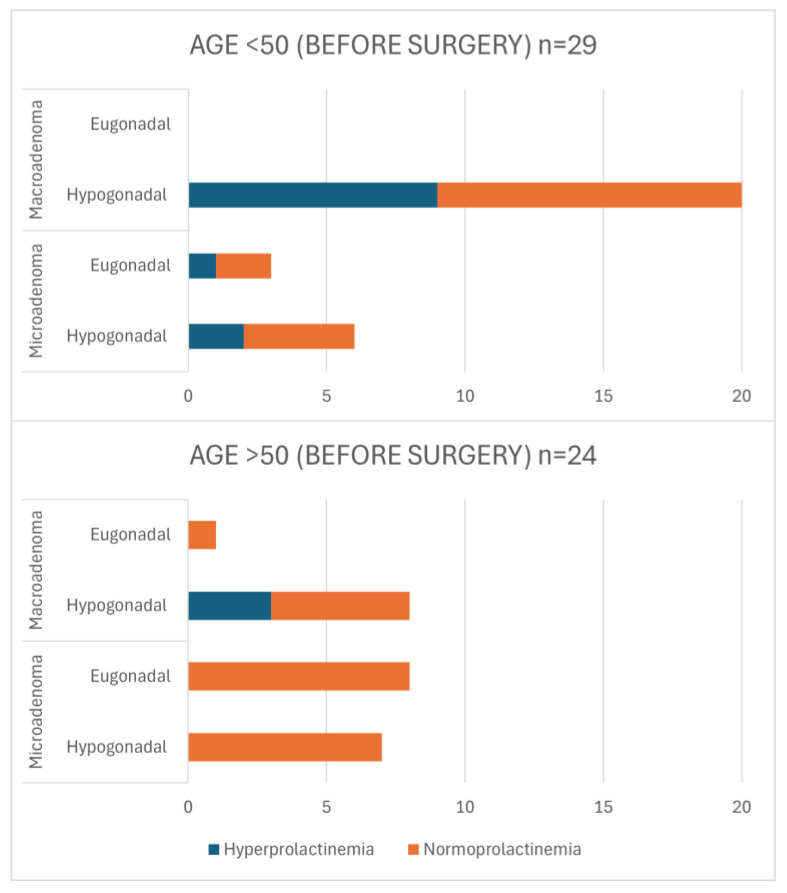
Characteristics of pre-operative patients in terms of age, tumor size, gonadal status, and presence of hyperprolactinemia in longitudinal sample. *p*-values for Chi-square test (<50 vs. >50): eu- vs. hypogonadism *p* = 0.019; micro- vs. macroadenoma *p* = 0.022; normo- vs. hyperprolactinemia *p* = 0.020.

Figure 4 outlines the pre-operative characteristics of patients in the longitudinal sample (detailed data of patients in this group are presented in Supplementary Table S3). Among the pre-operative cohort, hypogonadism was diagnosed in 41 males (77.36%). Most of the patients presented with macroadenoma. Overall, more than 35% of tumors invaded the cavernous sinus and optic chiasm compression was present in over 30% of pre-operative patients. For patients aged <50 years, almost 90% were hypogonadal. Almost 70% were diagnosed with macroadenomas, all of whom had hypogonadism. Hyperprolactinemia was present in 45% of these patients. In patients with microadenoma in this age group, 67% were diagnosed with hypogonadism and 33% demonstrated concurrent hyperprolactinemia. In the >50 years age group, only 37.5% of patients were diagnosed with macroadenomas, with 89% of them presenting hypogonadism. Hyperprolactinemia was identified in 38% of these patients. A total of 47% of patients with microadenoma presented with hypogonadism. The comparison between both age groups showed a significant difference in the presence of hypogonadism (*p* = 0.019). Similarly, significant differences were found between microadenomas and macroadenomas (*p* = 0.022), and between normoprolactinemia and hyperprolactinemia (*p* = 0.020).

**Figure 5:**
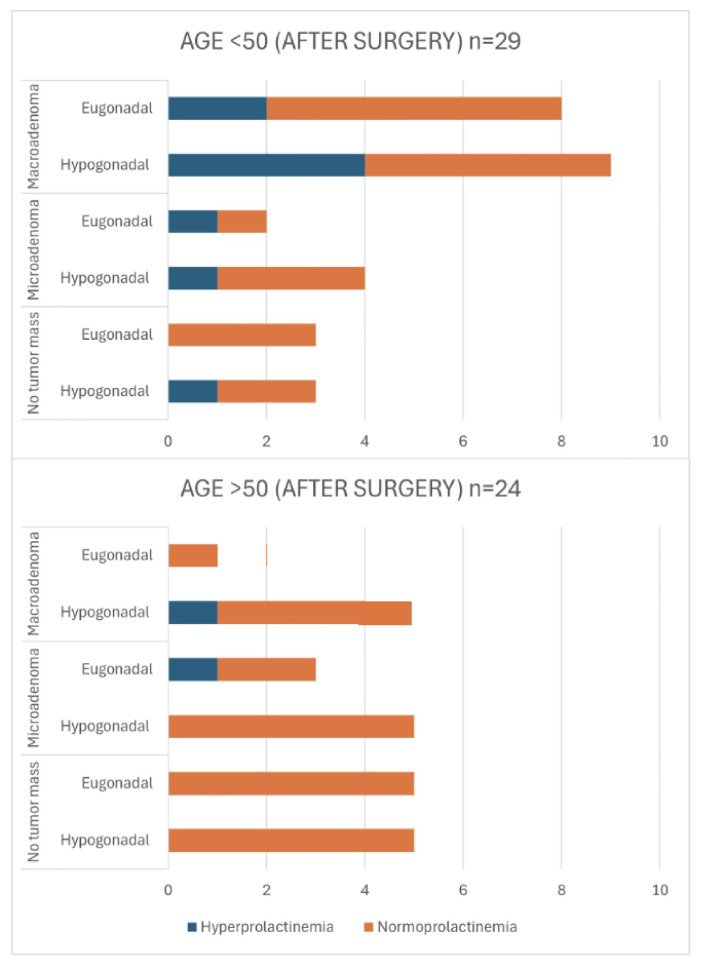
Characteristics of post-operative patients in terms of age, tumor size, gonadal status, and presence of hyperprolactinemia in longitudinal sample. *p*-values for Chi-square test (<50 vs. >50): eu- vs. hypogonadism *p* = 0.817; micro- vs. macroadenoma *p* = 0.014; normo- vs. hyperprolactinemia *p* = 0.043.

Figure 5 presents an overview of post-operative outcomes in patients during the follow-up period (detailed data on these outcomes are presented in Supplementary Table S4). Overall, the prevalence of hypogonadism decreased to 58.49%, with 31 individuals affected. In patients <50 years, the prevalence of hypogonadism decreased up to 55%, and hyperprolactinemia was present in 37.5% of them. In patients with no tumor mass, three out of six had exhibited ongoing hypogonadism. Within patients with residual tumor mass, hypogonadism was present in 67% of patients with microadenoma, and 53% of those with macroadenoma. In the older age group (>50 years), hypogonadism was present in 58%. Among individuals with macroadenomas, five out of six patients presented persistent hypogonadism post-surgery. The comparison between eugonadism and hypogonadism showed no statistically significant difference between the age groups (*p* = 0.817). However, significant differences were observed when comparing microadenomas to macroadenomas (*p* = 0.014) and normoprolactinemia to hyperprolactinemia (*p* = 0.043).

#### 3.2.1. Effect of Neurosurgical Treatment

Postoperative testosterone concentrations increased significantly (9.1 vs. 12.1 nmol/L; *p* < 0.001), particularly in patients with preoperative hypogonadism (7.2 vs. 10.2 nmol/L; *p* < 0.001). Gonadotropin levels postoperatively demonstrated a significant elevation (LH: 3.4 vs. 3.9 mlU/mL; *p* = 0.007; FSH: 4.9 vs. 6.1 mlU/mL; *p* = 0.032), especially in patients with preoperative hypogonadism (LH: 2.7 vs. 3.2 mlU/mL; *p* = 0.003; FSH: 4.3 vs. 6.05 mlU/mL; *p* = 0.032). We observed a significant elevation of SHBG in patients with preoperative hypogonadism (22.6 vs. 27.4 nmol/L; *p* = 0.012). Changes in FTI were not significant. GH and IGF-1 concentrations postoperatively decreased (GH: 2.62 vs. 1.52 ng/mL; *p* = 0.002; IGF-1: 498 vs. 290 ng/mL; *p* < 0.001). Patients with preoperative hypogonadism presented higher median baseline values of GH and IGF-1 (GH: 3.37 ng/mL; IGF-1: 551 ng/mL) compared to those without hypogonadism (GH: 1.36 ng/mL; IGF-1: 355 ng/mL). Total cholesterol and low-density lipoprotein (LDL) values experienced a statistically significant reduction postoperatively across all cohorts (*p* < 0.001). We observed higher baseline values of LDL and triglycerides in patients with preoperative hypogonadism. The level of phosphorus was higher in patients with preoperative HH (3.95 vs. 3.51) and decreased significantly after surgery in both groups. A decrease in SAGIT scores was observed postoperatively (7 vs. 6; *p* < 0.001). Nevertheless, baseline and postoperative values were higher in the HH group. Detailed results are presented in Table 3.

Table 4 presents the outcomes of neurosurgical treatment. Of the 41 patients with hypogonadism initially, we observed recovery in 12 patients (29.27%). In two patients, hypogonadism appeared after surgery. The exact Fisher’s test yield: *p* = 0.00174.

#### 3.2.2. Predictive Markers for Remission of Hypogonadism

**Figure 6:**
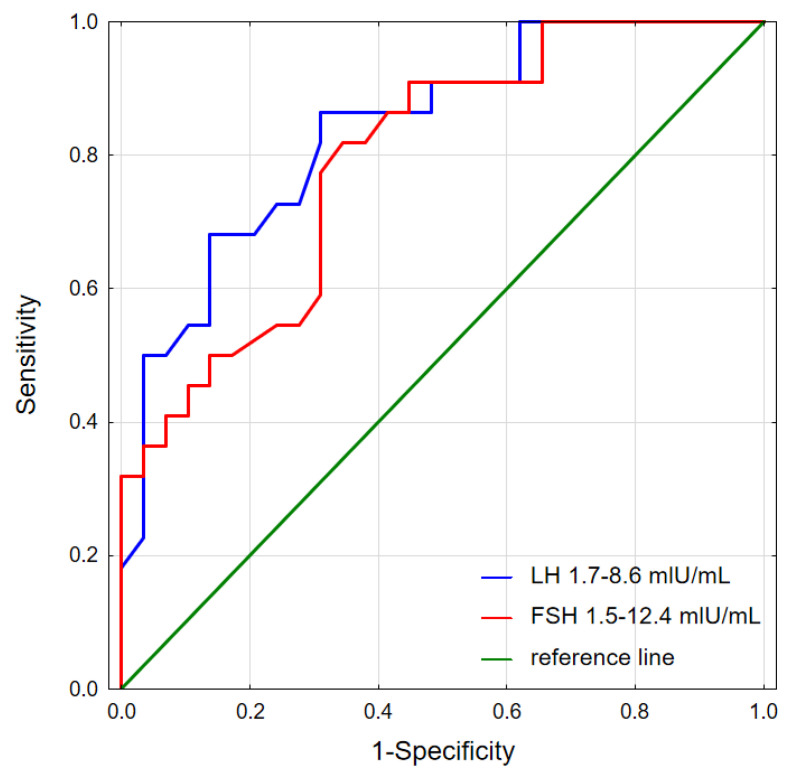
ROC curves for LH and FSH discriminating the remission of hypogonadism. LH and FSH predictive values were observed for discriminating the remission of hypogonadism with AUC = 0.838, cut-off was 3.3 mIU/mL and 0.792, cut-off was 4.4 mIU/mL.

We performed ROC analysis and showed that we can predict the remission of hypogonadism based on preoperative LH and FSH values in longitudinal group (Figure 6). LH higher than 3.3 mIU/mL and FSH higher than 4.4 mIU/mL predicted the remission of hypogonadism with AUC = 0.838, and 0.792, respectively.

### 3.3. Relationships of Pre- and Postoperative Parameters

Serum testosterone significantly correlated with gonadotropins levels, prolactin, SHBG, and FTI scores in patients with hypogonadism before surgery (Table 5).

We also correlated serum testosterone and metabolic parameters. Serum testosterone inversely correlated with triglycerides (TG) in patients with HH after surgery. We did not find correlations with total cholesterol (TC), low-density lipoprotein (LDL), high-density lipoprotein (HDL), and glucose in this group. LDL, TG, and glucose levels correlated negatively with testosterone in patients without hypogonadism (Table 6).

We found that hyperprolactinemia and larger tumor size (macroadenoma) was associated with HH (Table 7).

## 4. Discussion

We aimed to investigate the incidence of hypogonadism among men diagnosed with acromegaly and to evaluate the impact of neurosurgical intervention during the follow-up period. Among the pre-operative cohort hypogonadism was diagnosed in more than three-quarters of them, with prevalence being higher in younger patients with macroadenomas. In the longitudinal sample, following surgery, every third patient recovered from HH and the prevalence of hypogonadism decreased to less than 60%. As expected, testosterone levels were significantly lower in patients with macroadenoma compared to microadenoma, and hypogonadism was manifested more frequently in younger patients (<50 years). We revealed a noteworthy association between testosterone levels, age, and adenoma size. Our findings indicate a higher predisposition to pre-operative hypogonadism in younger patients diagnosed with macroadenoma, and hyperprolactinemia. We also found a clear association between surgical intervention and the decrease in hypogonadism prevalence. Our findings reveal that patients with pre-operative values of LH > 3.3 mIU/mL and FSH > 4.4 mIU/mL had higher chance for remission of hypogonadism.

Patients presenting with preoperative hypogonadism exhibited elevated baseline concentrations of GH and IGF-1. High GH and IGF-1 disrupt the typical pulsatile secretion of gonadotropins at the hypothalamic–pituitary level, leading to hypogonadotropic hypogonadism. Postoperative analyses unveiled a significant increase in testosterone and gonadotropin concentrations, particularly in individuals with preoperative hypogonadism. Although HH, either isolated or with other hormonal abnormalities, can result from surgical or radiation therapy, we observed this complication only in two patients. According to the literature, hypopituitarism is a common complication of surgery for PA, which affects one-third of patients within three months [20,21]. However, complications in patients with acromegaly appear to be less frequent in comparison to patients who underwent transsphenoidal surgery (TSS) for another benign pituitary tumor (0.3% vs. 1.1%; *p* < 0.05) [22]. This underscores the role of surgical intervention, in reducing the prevalence of hypogonadism.

Most of the patients presented with macroadenoma. We found HH in more than 90% of cases. In addition, macroadenomas were more common in younger patients (<50 years of age). The association between larger and more invasive tumors and hypogonadism further highlights the influence of tumor size on pituitary function and architecture. Interestingly, within the group of patients with pituitary macroadenomas, the incidence of hypogonadism is notably higher in those with functional PA than with non-functional PA [23]. We also confirmed that in CG with NFPA only 15% of patients presented HH. The presence of a pituitary macroadenoma can inhibit gonadotropic function through direct compression of gonadotropic cells or by causing deviation in the pituitary stalk, leading to hyperprolactinemia. In some cases, this hyperprolactinemia results directly from co-secretion by the adenoma itself. Furthermore, as mentioned earlier, elevated levels of growth hormones (GH) and insulin-like growth factor 1 (IGF-1), regardless of the tumor size, can disrupt the normal pulsatility of gonadotropin secretion [4]. Elevated prolactin levels lead to a decrease in the frequency and amplitude of LH pulses. However, normalization of serum prolactin can reverse the suppression of LH pulses [24,25]. Pre-surgery hyperprolactinemia was found in 37.5% of patients with hypogonadism, including 51.6% of patients with macroadenoma. Although gonadotropin levels were found to be comparable between patients with microadenomas and macroadenomas, the incidence of hypogonadism was more pronounced in the latter group. This observation suggests that hyperprolactinemia may exert an additional inhibitory effect on the hypothalamic–pituitary–gonadal axis, beyond what is reflected in gonadotropin levels alone. Importantly, no linear relationship was observed between the levels of tropic hormones and their respective effector hormones. The comparable gonadotropin levels across groups may also be attributed to the suppressive action of elevated PRL, particularly on LH secretion. This suppressive effect is likely exacerbated by the higher PRL levels typically associated with larger tumor size. This finding is supported by data from the Pituitary Tumor Registry, according to which 49% of male patients with acromegaly experience HH, and 45% of them have concurrent hyperprolactinemia [23,26]. The normalization of prolactin levels after surgery, especially with the concurrent normalization of GH and IGF-1, directly affects testosterone levels. SHBG production is inhibited by insulin, prolactin, and GH. SHBG plasma levels are altered in acromegaly. We observed lower SHBG in patients with larger tumors and higher GH. Consequently, although total testosterone was lower in macroadenomas compared to microadenomas, free testosterone levels remain comparable. The hypothalamic–pituitary–gonadal (HPG) axis is crucial for spermatozoa production and the preservation of sexual function in men [23]. The observed impaired testicular function, abnormal sperm production, and diminished serum testosterone levels in male acromegaly patients diagnosed with hypogonadism highlight the clinical consequences, including male infertility, increased BMI, reduced physical strength, lowered libido, loss of morning erections, and erectile dysfunction [4]. LH is responsible for regulating testosterone production in Leydig cells. Simultaneously, FSH collaborates with testosterone, regulating the production of essential regulatory molecules and nutrients crucial for the maturation of spermatogonia into sperm cells [27]. The surgical removal of PA not only relieves compression on normal pituitary tissue but also reinstates regular pituitary function by eliminating the excess GH and IGF-1 that disrupts FSH/LH secretion. As mentioned earlier, the regular pulsatility of LH and FSH secretion at the hypothalamic–pituitary level is altered due to elevated GH and IGF-1, resulting in a state of hypogonadotropic hypogonadism irrespective of adenoma size.

Although gonadal dysfunction is quite a common problem in patients with acromegaly, there is still little data on male fertility. In a study comprising 35 patients aged 27 to 59 years with active disease and an equal number of age-matched healthy controls, gonadal hormones and seminal fluid analysis were assessed both before and 6 months after surgery or lanreotide administration. After treatment, all patients had a significant rise in T, while only those with controlled disease reported an increase in gonadotropins. Similarly, an increase in sperm count was found in all patients, while motility was significantly increased only in patients with controlled disease [28]. Another study found that although patients with acromegaly had significantly reduced T levels, they showed no differences in semen volume, sperm count, and sperm motility compared to the healthy subjects [29]. These data may indicate a stimulating effect of GH/IGF-I activity on sperm motility. Due to conflicting results, further studies are needed in this subject.

Due to chronic exposure to GH and IGF-1, patients with acromegaly are at a higher risk of CVD, certain neoplasms, obstructive sleep apnea, arthropathy, as well as endocrine and metabolic disorders [16,30,31,32]. These comorbidities notably impair quality of life and also decrease life expectancy [33]. Male hypogonadism is usually associated with low HDL-C, and increased LDL-C and TG [34]. In our study, we observed a significant decrease in total cholesterol and LDL values postoperatively in the longitudinal sample. Baseline values of LDL and triglycerides were higher in patients with preoperative hypogonadism. Results from studies researching lipid profiles in patients with acromegaly report total cholesterol to be increased, normal or even decreased [35]. Triglycerides seem to be mostly elevated, while high-density lipoprotein cholesterol levels are typically low [34,35]. Lipid metabolism abnormalities in acromegaly are closely connected to glucose homeostasis disruptions. Some effects are directly caused by GH, while others are mediated by IGF-1 [36]. GH promotes lipolysis in adipose tissue, increasing the release of free fatty acids (FFAs) into the bloodstream, which fuels beta-oxidation and ketogenesis. Elevated circulating FFAs contribute to the formation of triglyceride-rich, very low-density lipoproteins (VLDL) and play a major role in the insulin resistance associated with acromegaly. The most common lipid abnormalities in acromegaly patients include hypertriglyceridemia and reduced HDL-cholesterol levels [37]. We observed significantly lower HDL levels in patients with acromegaly than in CG with NFPA; however, both were within the norm range. One of the primary metabolic effects of GH excess is reduced peripheral glucose uptake coupled with increased glucose production by the liver. GH excess disrupts normal insulin receptor phosphorylation and its signaling pathways, leading to insulin resistance [38]. Diabetes mellitus is observed in 20–53% of individuals with acromegaly, while the prevalence of impaired fasting glucose ranges from 8.9–19% and glucose intolerance from 15–31.6% [36]. We observed diabetes in over 30% of patients, and fasting glucose levels were significantly higher in patients with acromegaly than those with NFPA.

Although there is a limited number of papers available comparing the incidence of hypogonadism pre- and postoperatively, our study had certain limitations. We were using a retrospective study design, and due to the lack of follow-up with some patients, we decided to present the data in two groups. Patients from the longitudinal group were also included in the cross-sectional sample—this way we were able to compare a larger number of the patients. In addition, Liquid Chromatography–Tandem Mass Spectrometry (LC-MS/MS) is considered the gold standard and the most precise method for evaluating sex steroids. Nonetheless, standardized automated platform immunoassays for total testosterone assessment show a strong correlation with LC-MS/MS. This study only represented the patient population from single facility in Poznan, Poland. The cut-off of GH in 75 g OGTT was <1 ng/mL, since it is used in our laboratory. Subsequent investigations should aim to incorporate additional variables such as semen quality to further elucidate the multifaceted dynamics of this subject.

## 5. Conclusions

Younger patients with macroadenoma and hyperprolactinemia are more likely to have preoperative hypogonadism. Neurosurgical treatment can normalize not only GH and IGF-1 levels but also LH, FSH, and total testosterone. Every third male patient with acromegaly and hypogonadism can recover from HH after tumor resection, with a better chance of recovery for controlled acromegaly.

## Figures and Tables

**Table 1:** Clinical characteristics of patients pre- and post-surgery.

	Before Surgery		After Surgery		Control Group
Parameter	Cross-Sectional Sample N = 62	Longitudinal Sample N = 53	Cross-Sectional Sample N = 60	Longitudinal Sample N = 53	N = 60
Age (mean ± SD)	49.2 (±14.02)	49.3 (±13.3)	50.4 (±13.34)	49.6 (±13.4)	49.8 (±17.0)
<50 years	32 (51.61%)	29 (54.72%)	29 (48.33%)	29 (54.72%)	29 (48.33%)
>50 years	30 (48.39%)	24 (45.28%)	31 (51.77%)	24 (45.28%)	31 (51.67%)
Hypogonadism	48 (77.42%)	41 (77.36%)	36 (60.00%)	31 (58.49%)	9 (15.00%)
Hyperprolactinemia (N: 85–390 uIU/mL)	19 (30.65%)	15 (28.30%)	16 (26.67%)	11 (20.75%)	15 (25.00%)
Macroadenoma	34 (54.84%)	29 (54.72%)	25 (41.67%)	23 (43.40%)	33 (55.00%)
Invasive tumor	22 (35.48%)	19 (35.85%)	19 (31.67%)	17 (32.08%)	26 (43.33%)
Chiasmal compression	19 (30.65%)	18 (33.96%)	17 (28.33%)	16 (30.19%)	19 (31.67%)
BMI (mean ± SD)	29.9 (±5.82)	30.3 (±6.23)	30.41 (±5.85)	30.20 (±6.15)	30.10 (±4.49)
Prostatic hyperplasia	15 (24.19%)	14 (26.42%)	19 (31.67%)	14 (26.42%)	9 (15.00%)
<50 years	2 (13.33%)	2 (14.29%)	2 (10.53%)	2 (14.29%)	1 (11.11%)
>50 years	13 (86.67%)	12 (85.71%)	17 (89.47%)	12 (85.71%)	8 (88.89%)
Comorbidities					
Arterial hypertension	45 (72.58%)	39 (73.58%)	44 (73.33%)	39 (73.58%)	38 (63.33%)
Diabetes mellitus	19 (30.65%)	16 (30.19%)	18 (30.00%)	16 (30.19%)	14 (23.33%)
Obstructive sleep apnea	1 (1.61%)	1 (1.89%)	1 (1.67%)	1 (1.89%)	0
Nodular goiter	32 (51.61%)	30 (56.60%)	35 (58.33%)	30 (56.60%)	24 (40.00%)
Joint pain	10 (16.13%)	8 (15.10%)	10 (16.67%)	8 (15.10%)	5 (8.33%)
Osteoporosis	7 (11.29%)	6 (11.32%)	6 (10.00%)	6 (11.32%)	3 (5.00%)

BMI—body mass index, SD—standard deviation.

**Table 2:** Comparisons of laboratory parameters before neurosurgical treatment in patients with micro- and macroadenoma and control group (NFPA).

Parameter (Median)	Cross-Sectional		Control Group (NFPA)		*p*-Value
	Microadenoma [1]	Macroadenoma [2]	Microadenoma CG [1c]	Macroadenoma CG [2c]	
	n = 28	n = 34	n = 27	n = 33	
Testosterone [9.9–27.8 nmol/L]	11.05 ^•#^	7.2 ^•∇♦^	16.7 ^#∇^	13.1 ^♦^	<0.001 *
LH [1.7–8.6 mlU/mL]	3.1	3.9	3.6	3.7	0.557
FSH [1.5–12.4 mlU/mL]	4.65	5.7	5.9	6.0	0.523
PRL [85–390 uIU/mL]	202 ^•^	304 ^•^	254	292	0.033 *
SHBG [14.5–48.4 nmol/L]	31.7	22.6 ^∇♦^	43.5 ^∇^	38.5 ^♦^	<0.001 *
FTI [33.8–106%]	33.01	33.01	36.44	34.58	0.375
TSH [0.27–4.20 μU/mL]	0.79 ^#■^	1.31	1.65 ^#^	1.91 ^■^	0.002 *
fT4 [11.5–21.0 pmol/L]	15.26	15.9	15.23	14.00	0.130
HBG [13.5–17.2 g/dL]	14.65	14.9	15.2	14.5	0.982
HCT [39.5–50.5%]	42.35	42.9	43.1	42.8	0.445
Glucose [70–99 mg/dL]	101.5	109 ^∇♦^	95 ^∇^	95 ^♦^	<0.001 *
GH [0.03–2.47 ng/mL]	2.12 ^#■^	5.73 ^∇♦^	0.20 ^#∇^	0.20 ^♦■^	<0.001 *
IGF1 [ng/mL]	456.5 ^#■^	702 ^∇♦^	132 ^#∇^	111 ^♦■^	<0.001 *
TC [<190 mg/dL]	198	201	194	194	0.895
LDL [<115 mg/dL]	105.05	125	132	130	0.102
HDL [>35 mg/dL]	56	50 ^∇♦^	61 ^∇^	64 ^♦^	0.014 *
TG [<150 mg/dL]	108.5	129	122	123	0.082
Phosphorus [2.70–4.50 mg/dL]	3.69	4.08 ^∇♦^	3.33 ^∇^	3.33 ^♦^	<0.001 *

LH—luteinizing hormone; FSH—follicle-stimulating hormone; PRL—prolactine; SHBG—sex hormone binding globulin; FTI—free testosterone index; TSH—thyroid-stimulating hormone; fT4—free thyroxine; HBG—hemoglobin; HCT—hematocrit; GH—growth hormone; IGF1—insulin-like growth factor 1; TC—total cholesterol; LDL—low-density lipoprotein; HDL—high-density lipoprotein; TG—triglicerides; *—significant differences for *p*-value < 0.05; n/s—non-significant; CG—control group; post-hoc significance between groups: • (1 vs. 2); # (1 vs. 1c); ■ (1vs. 2c); ∇ (2 vs. 1c); ♦ (2 vs. 2c).

**Table 3:** Comparisons of laboratory parameters before and after neurosurgical treatment in longitudinal sample.

Median	All			Preoperative HH			No Preoperative HH		
	Before	After	*p*-Value	Before	After	*p*-Value	Before	After	*p*-Value
Testosterone [9.9–27.8 nmol/L]	9.1	12.1	<0.001 *	7.2	10.2	<0.001 *	14.6	15.95	0.374
LH [1.7–8.6 mlU/mL]	3.4	3.9	0.007 *	2.7	3.2	0.003 *	5.4	4.75	0.575
FSH [1.5–12.4 mlU/mL]	4.9	6.1	0.032 *	4.3	6.05	0.032 *	5.45	6.1	0.554
PRL [85–390 uIU/mL]	234	189	0.051	258	231	0.066	190	165	0.480
SHBG [14.5–48.4 nmol/L]	26	29.1	0.007 *	22.6	27.4	0.012 *	47	41.7	0.686
FTI [33.8–106%]	33.01	34.34	0.353	33.01	32.86	0.263	36.2	35.43	0.686
TSH [0.27–4.20 μU/mL]	1.3	0.92	0.016 *	1.31	0.7	0.015 *	0.82	0.98	0.784
fT4 [11.5–21.0 pmol/L]	15.97	17.21	0.03 *	15.9	17.64	0.018 *	16.14	15.4	0.814
HBG [13.5–17.2 g/dL]	14.9	14.5	0.048 *	14.6	14.5	0.141	15.25	14.8	0.069
HCT [39.5–50.5%]	42.2	42.4	0.706	42	42.2	0.862	43.45	43.5	0.575
Glucose [70–99 mg/dL]	103	104	0.114	103	104	0.401	103	103	
GH [0.03–2.47 ng/mL]	2.62	1.52	0.002 *	3.37	1.53	0.001 *	1.36	1.118	0.499
IGF1 [ng/mL]	498	290	<0.001 *	551	329	<0.001 *	355	215	0.012 *
TC [<190 mg/dL]	199.5	182.5	<0.001 *	193.5	183	<0.001 *	200.5	180.5	0.018 *
LDL [<115 mg/dL]	122.4	106	<0.001 *	123	106	0.005 *	112	114	0.028 *
HDL [>35 mg/dL]	52	48	0.813	50	49	0.602	59	47.5	0.917
TG [<150 mg/dL]	112.5	104	0.165	124.5	100	0.434	109	108	0.237
Phosphorus [2.70–4.50 mg/dL]	3.82	3.52	<0.001 *	3.95	3.7	0.005 *	3.51	3.12	0.028 *
SAGIT	7	6	<0.001 *	9	8	<0.001 *	5.5	5	0.012 *
BMI [kg/m^2^]	28.9	28.4	0.313	29.6	29.4	0.266	27.5	27.1	>0.999

HH—Hypogonadotropic hypogonadism; LH—luteinizing hormone; FSH—follicle-stimulating hormone; PRL—prolactine; SHBG—sex hormone binding globulin; FTI—free testosterone index; TSH—thyroid-stimulating hormone; fT4—free thyroxine; HBG—hemoglobin; HCT—hematocrit; GH—growth hormone; IGF1—insulin-like growth factor 1; TC—total cholesterol; LDL—low-density lipoprotein; HDL—high-density lipoprotein; TG—triglicerides; BMI—body mass index; *—significant differences for *p*-value < 0.05.

**Table 4:** Bivariate table with observed frequencies of hypogonadism and active acromegaly.

Pre-Surgery Hypogonadism	Bivariate Table with Observed Frequencies of Hypogonadism and Active Acromegaly		
	Post-Surgery Hypogonadism No	Post-Surgery Hypogonadism Yes	Total
**No (% column)**	10 (45.45%)	2 (6.45%)	12
**Yes (% column)**	12 (54.55%)	29 (93.55%)	41
**Total**	22	31	53
**No (%row)**	10 (83.33%)	2 (16.67)	12
**Yes (%row)**	12 (29.27%)	29 (70.73%)	41
**Total**	22	31	53

**Table 5:** Correlations between testosterone and LH, FSH, PRL, SHBG, and FTI in patients before and after neurosurgical treatment; with and without preoperative hypogonadism (Spearman’s rank correlation coefficients).

Parameter	Group	LH [mlU/mL]		FSH [mlU/mL]		PRL [uIU/mL]		SHBG [nmol/L]		FTI [%]	
Testosterone [nmol/L]		Rs	*p*-value	Rs	*p*-value	Rs	*p*-value	Rs	*p*-value	Rs	*p*-value
	All pre-surgery	0.423	0.001 *	0.229	0.086	−0.339	0.009 *	0.555	<0.001 *	0.353	0.0319 *
	All post-surgery	0.529	<0.001 *	0.397	0.002 *	−0.024	0.860	0.450	0.002 *	0.559	0.0001 *
	HH pre-surgery	0.407	0.006 *	0.370	0.013 *	−0.317	0.033 *	0.374	0.041 *	0.459	0.0139 *
	HH post-surgery	0.309	0.075	0.227	0.189	0.014	0.939	0.321	0.102	0.647	<0.001 *
	No HH pre-surgery	0.457	0.116	0.331	0.270	0.052	0.865	0.728	0.026 *	−0.176	0.651
	No HH post-surgery	0.328	0.136	0.014	0.952	−0.144	0.501	0.316	0.216	0.091	0.729

HH—hypogonadotropic hypogonadism; LH—luteinizing hormone; FSH—follicle-stimulating hormone; PRL—prolactin; SHBG—sex hormone binding globulin; FTI—free testosterone index; Rs—Spearman’s rank correlation coefficients; *—significant differences for *p*-value < 0.05.

**Table 6:** Correlations between testosterone and lipid profiles in patients before and after neurosurgical treatment; with and without preoperative hypogonadism (Spearman’s rank correlation coefficients).

Parameter	Group	TC [mg/dL]		LDL [mg/dL]		HDL [mg/dL]		TG [mg/dL]		Glucose [mg/dL]	
Testosterone [nmol/L]		Rs	*p*-value	Rs	*p*-value	Rs	*p*-value	Rs	*p*-value	Rs	*p*-value
	All pre-surgery	−0.261	0.124	−0.122	0.519	0.249	0.144	−0.424	0.010	−0.061	0.644
	All post-surgery	−0.166	0.259	−0.201	0.181	0.356	0.013 *	−0.544	0.000 *	−0.158	0.228
	HH pre-surgery	−0.331	0.085	−0.007	0.976	0.069	0.726	−0.316	0.101	0.069	0.650
	HH post-surgery	−0.029	0.882	0.108	0.584	0.286	0.140	−0.568	0.002 *	0.253	0.137
	No HH Pre-surgery	−0.587	0.126	−0.886	0.019 *	0.707	0.050 *	−0.910	0.002 *	−0.687	0.009 *
	No HH post-surgery	−0.244	0.299	−0.569	0.014 *	0.458	0.042 *	−0.614	0.005 *	−0.485	0.016 *

HH—Hypogonadotropic hypogonadism; TC—total cholesterol; LDL—low-density lipoprotein; HDL—high-density lipoprotein; TG—triglycerides; Rs—Spearman’s rank correlation coefficients; *—significant differences for *p*-value < 0.05.

**Table 7:** Associations between hypogonadism and comorbidities, age, and tumor size (Pearson’s chi-squared test).

	Pre-Surgery Hypogonadism	Post-Surgery Hypogonadism
	*p*-Value	*p*-Value
Hyperprolactinemia	0.027 *	0.378
Prostatic hyperplasia	0.925	0.572
Obesity	0.33	0.092
Diabetes melitus	0.395	0.206
Age > 50	0.136	>0.999
Tumor size—categorized	0.025 *	0.44

*—significant differences for *p*-value < 0.05.

## Data Availability

The datasets generated during and/or analyzed during the current study are available from the corresponding author on reasonable request.

## Supplementary Materials

The following supporting information can be downloaded at: https://www.mdpi.com/article/10.3390/jcm13185526/s1, Table S1: Detailed characteristics of pre-operative patients in terms of age, tumor size, gonadal status, and presence of hyperprolactinemia in cross-sectional sample; Table S2: Detailed characteristics of post-operative patients in terms of age, tumor size, gonadal status, and presence of hyperprolactinemia in cross-sectional sample; Table S3: Detailed characteristics of pre-operative patients in terms of age, tumor size, gonadal status, and presence of hyperprolactinemia in longitudinal sample; Table S4: Detailed characteristics of post-operative patients in terms of age, tumor size, gonadal status, and presence of hyperprolactinemia in longitudinal sample.
