# Supplementary material for: Every Third Male Patient with Acromegaly Recovers from Hypogonadism after Neurosurgical Treatment
Source: J Clin Med. 2024 Sep 18;13(18):5526. doi: 10.3390/jcm13185526 (PMC11432164; doi:10.3390/jcm13185526)
Supplement: Supplementary file 1 [file jcm-13-05526-s001.zip › jcm-3148233-supplementary.pdf]

<u>Supplementary material</u>

Supplementary Table 1. Detailed characteristics of pre-operative patients in terms of age, tumor size, gonadal status, and presence of hyperprolactinemia in cross-sectional sample.

AGE <50 (BEFORE SURGERY) n=32						
	Microadenoma			Macroadenoma		
Gonadal status	Hyperprolactinemia	Normoprolactinemia	Total	Hyperprolactinemia	Normoprolactinemia	Total
Hypogonadal	2 (25%)	6 (75%)	8 (73%)	9 (47%)	10 (53%)	19 (90%)
Eugonadal	1 (33%)	2 (77%)	3 (27%)	0 (0%)	2 (100%)	2 (10%)
Total	3 (27%)	8 (73%)	11 (100%)	9 (43%)	12 (57%)	21 (100%)
AGE >50 (BEFORE SURGERY) n=30						
	Microadenoma			Macroadenoma		
Gonadal status	Hyperprolactinemia	Normoprolactinemia	Total	Hyperprolactinemia	Normoprolactinemia	Total
Hypogonadal	0 (0%)	9 (100%)	9 (100%)	7 (58%)	5 (42%)	12 (92%)
Eugonadal	0 (0%)	8 (100%)	8 (100%)	0 (0%)	1 (100%)	1 (8)
Total	0 (0%)	17 (100%)	17 (100%)	7 (54%)	5 (46%)	13 (100%)

Supplementary table 2. Detailed characteristics of post-operative patients in terms of age, tumor size, gonadal status, and presence of hyperprolactinemia in cross-sectional sample.

AGE <50 (AFTER SURGERY) n=29											
No tumor mass				Microadenoma				Macroadenoma			
Gonadal status	Hyperprolactinemia	Normoprolactinemia	Total	Gonadal status	Hyperprolactinemia	Normoprolactinemia	Total	Gonadal status	Hyperprolactinemia	Normoprolactinemia	Total
Hypogonadal	1 (33%)	2 (67%)	3 (50%)	Hypogonadal	1 (25%)	3 (75%)	4 (67%)	Hypogonadal	5 (50%)	5 (50%)	10 (59%)
Eugonadal	0 (0%)	3 (100%)	3 (50%)	Eugonadal	1 (50%)	1 (50%)	2 (33%)	Eugonadal	5 (71%)	2 (29%)	7 (41%)
Total	1 (17%)	5 (83%)	6 (10	Total	2 (33%)	4 (67%)	6 (100	Total	10 (59%)	7 (41%)	17 (100

			0%)				%)				%)
AGE >50 (AFTER SURGERY) n=31											
No tumor mass				Microadenoma				Macroadenoma			
Gonadal status	Hyperprolactinemia	Normoprolactinemia	Total	Gonadal status	Hyperprolactinemia	Normoprolactinemia	Total	Gonadal status	Hyperprolactinemia	Normoprolactinemia	Total
Hypogonadal	1 (13%)	7 (87%)	8 (62%)	Hypogonadal	0 (0%)	6 (100%)	6 60%)	Hypogonadal	1 (20%)	4 (80%)	5 (63%)
Eugonadal	0 (0%)	5 (5%)	5 (38%)	Eugonadal	1 (25%)	3 (75%)	4 (40%)	Eugonadal	0 (0%)	3 (100%)	3 (37%)
Total	1 (8%)	12 (92%)	13 (100%)	Total	1 (10%)	9 (90%)	10 (100%)	Total	1 (13%)	7 (87%)	8 (100%)

Supplementary table 3. Detailed characteristics of pre-operative patients in terms of age, tumor size, gonadal status, and presence of hyperprolactinemia in longitudinal sample.

AGE <50 (BEFORE SURGERY) n=29						
	Microadenoma			Macroadenoma		
Gonadal status	Hyperprolactinemia	Normoprolactinemia	Total	Hyperprolactinemia	Normoprolactinemia	Total
Hypogonadal	2 (33%)	4 (67%)	6 (67%)	9 (45%)	11 (55%)	20 (100%)
Eugonadal	1(33%)	2 (67%)	3 (33%)	0 (0%)	0 (0%)	0 (0%)
Total	3 (33%)	6 (67%)	9 (100%)	9 (45%)	11 (55%)	20 (100%)
AGE >50 (BEFORE SURGERY) n=24						
	Microadenoma			Macroadenoma		
Gonadal status	Hyperprolactinemia	Normoprolactinemia	Total	Hyperprolactinemia	Normoprolactinemia	Total
Hypogonadal	0 (0%)	7 (100%)	7 (47%)	3 (38%)	5 (62%)	8 (89%)
Eugonadal	0 (0%)	8 (100%)	8 (53%)	0 (0%)	1 (100%)	1 (11%)
Total	0 (0%)	15 (100%)	15 (100%)	3 (33%)	6 (67%)	9 (100%)

Supplementary table 4. Detailed characteristics of post-operative patients in terms of age, tumor size, gonadal status, and presence of hyperprolactinemia in longitudinal sample.

AGE <50 (AFTER SURGERY) n=29											
No tumor mass				Microadenoma				Macroadenoma			
Gonadal status	Hyperprolactinemia	Normoprolactinemia	Total	Gonadal status	Hyperprolactinemia	Normoprolactinemia	Total	Gonadal status	Hyperprolactinemia	Normoprolactinemia	Total
Hypogonadal	1 (33%)	2 (67%)	3 (50%)	Hypogonadal	1 (25%)	3 (75%)	4 (67%)	Hypogonadal	4 (44%)	5 (66%)	9 (53%)
Eugonadal	0 (0%)	3 (100%)	3 (50%)	Eugonadal	1 (50%)	1 (50%)	2 (33%)	Eugonadal	2 (25%)	6 (75%)	8(47%)
Total	1 (17%)	5 (83%)	6 (100%)	Total	2 (33%)	4 (67%)	6 (100%)	Total	6 (35%)	11 (65%)	17 (100%)
AGE >50 (AFTER SURGERY) n=24											
No tumor mass				Microadenoma				Macroadenoma			
Gonadal status	Hyperprolactinemia	Normoprolactinemia	Total	Gonadal status	Hyperprolactinemia	Normoprolactinemia	Total	Gonadal status	Hyperprolactinemia	Normoprolactinemia	Total
Hypogonadal	0 (0%)	5 (100%)	5 (50%)	Hypogonadal	0 (0%)	5 (100%)	5 (63%)	Hypogonadal	1 (20%)	4 (80%)	5 (83%)
Eugonadal	0 (0%)	5 (100%)	5 (50%)	Eugonadal	1 (33%)	2 (67%)	3 (37%)	Eugonadal	0 (0%)	1 (100%)	1 (17%)
Total	0 (0%)	10 (100%)	10 (100%)	Total	1 (13%)	7 (87%)	8 (100%)	Total	1 (17%)	5 (83%)	6 (100%)
